# Antimicrobial activities of pomegranate rind extracts: enhancement by addition of metal salts and vitamin C

**DOI:** 10.1186/1472-6882-8-64

**Published:** 2008-12-15

**Authors:** Erin M McCarrell, Simon WJ Gould, Mark D Fielder, Alison F Kelly, Waffa El Sankary, Declan P Naughton

**Affiliations:** 1 School of Life Sciences, Kingston University, Kingston-upon-Thames, UK

## Abstract

**Background:**

*Punica granatum *L. or pomegranates, have been reported to have antimicrobial activity against a range of Gram positive and negative bacteria. Pomegranate formulations containing ferrous salts have enhanced although short-term, antibacteriophage activities which are rapidly diminished owing to instability of the ferrous combination. The aim of this study was to determine the antimicrobial activities of combinations of pomegranate rind extracts (PRE) with a range of metals salts with the added stabiliser vitamin C.

**Methods:**

PRE solutions, prepared by blending rind sections with distilled water prior to sterilisation by autoclaving or filtration, were screened with a disc diffusion assay using penicillin G as a control. Suspension assays were used to determine the antimicrobial activities of PRE alone and in combination with salts of the following metals; Fe (II), Cu (II), Mn (II) or Zn (II), and vitamin C, against a panel of microbes following exposure for 30 mins. The test organisms included *Staphylococcus aureus*, *Bacillus subtilis, Escherichia coli, Pseudomonas aeruginosa *and *Proteus mirabilis*.

**Results:**

The screening assay demonstrated that PRE exhibited activity against the Gram positive organisms at 24 h with no observable effect on any of the Gram negative bacteria. However, after 12 h, zones of inhibition were only observed for *Ps. aeruginosa*. In contrast, using the suspension assay, addition of Cu (II) salts to PRE solutions extended the activities resulting in no detectable growth being observed for the PRE/Cu (II) combination against *E. coli, Ps. aeruginosa *and *P. mirabilis*. Minimal antimicrobial activity was observed following incubation with Fe (II), Mn (II) or Zn (II) salts alone or in combination with PRE against any of the organisms in the test panel. The addition of vitamin C markedly enhanced the activities of both PRE/Fe (II) and PRE/Cu (II) combinations against *S. aureus*.

**Conclusion:**

This is the first report demonstrating the enhanced efficacy of PRE/metal salt combinations in the presence of the stabilising agent vitamin C, to which all isolates were sensitive with the exception of *B. subtilis*. This study has validated the exploration of PRE along with additives such as metal salts and vitamin C as novel antimicrobial combinations.

## Background

Antimicrobial drug resistance in human bacterial pathogens is a continuing worldwide issue and as a consequence, effective treatment and control of such organisms remains an important challenge. Bacterial resistance has appeared for every major class of antibiotic [1]. Since their introduction the emergence of resistance to antibiotics has become increasingly evident, particularly for important pathogens such as *Escherichia coli *(*E. coli*), *Salmonella *spp., *Campylobacter *spp., *Enterococcus *spp. and *Staphylococcus *spp. [2,3].

Over the last decade research into the antimicrobial properties of traditional plant based medicines has been revisited [4-7]. Numerous plants have been screened for antimicrobial properties, for example Holetz and colleagues [5] tested 13 plants used in Brazilian traditional medicine and they demonstrated activity against bacteria such as *Staphylococcus aureus *(*S. aureus*) and *E. coli*. Meléndez *et al*., [7] tested 172 plant species used in Puerto Rico and they demonstrated that 14 of these showed activity against bacteria including *S. aureus *and *E. coli*.

*Punica granatum *L. (Punicaceae) referred to in English as pomegranates, have been highlighted in many studies as having antimicrobial activity against a range of both Gram positive and negative bacteria [4-7]. Prashanth and colleagues [8] tested a number of extracts of pomegranates against a range of bacteria (*S. aureus, E. coli, Klebsiella pneumoniae, Proteus vulgaris, Bacillus subtilis *(*B. subtilis*) and *Salmonella typhi*), and they found activity against all isolates. Braga and colleagues [9] observed that pomegranate extracts were able to inhibit not only the growth of *S. aureus *but also the production of enterotoxin. The methanolic extract derived from 200 g of dried pomegranate produced bactericidal effects at 1% (v/v) over an extended incubation period (50 hours), demonstrating longevity of action.

Many bacteria have advanced protective mechanisms for the detoxification of heavy metal ions [10]. Despite this, numerous literature reports address the development of metal compounds as antimicrobial agents. Many low-molecular-mass metal compounds exhibit bactericidal and/or bacteriostatic activities. In one study the susceptibilities of *Staphylococcus *strains to solutions of metal salts (in the range of 50 μmol to 80 mmol) were determined and frequencies of resistance were found to be CuSO_4 _and NiCl_2_, 36.2%; ZnSO_4_, 13.6% and CoCl_2_, 4.5% respectively [11]. In addition, all strains were sensitive to AgNO_3 _over the concentration range studied. Ionic silver salts have been particularly useful as a bactericide at minute concentrations. These apparent antimicrobial effects of metal ions coupled to a lack of significant toxicity in human cells has led to their incorporation into a wide range of healthcare products from catheters to wound dressings [12,13]. New metallo-antibiotic agents include a range of ligands that have been chelated to metal ions and to date, antimicrobial activities have been demonstrated for metal complexes of imidazoles, phenanthrolines, quinolones, aminoquinolines and benzoylhydrazones [14-17]. Despite the growing need for new antimicrobial therapies, the mechanisms of action of many metal binding antibiotics are not fully understood [18].

The enhancement of the antimicrobial activities exhibited by pomegranate rind extracts by the addition of metal ions has been investigated by Stewart *et al. *[19]. Their aim was to develop a rapid screening method for the detection of tuberculosis. They demonstrated that short term exposure to a pomegranate rind extract (PRE)/ferrous salt combination for 3 minutes reduced bacteriophage levels with no effect upon the bacteria. This short duration of exposure, although effective for the bacteriophage assay, was necessary owing to the low stability observed for the PRE/ferrous salt solutions.

The aim of this study was to enhance the activity of the PRE combination through modification of the formula by adding the stabilizer vitamin C. Enhancing the stability is intended to allow an increase in the exposure period to extend the antimicrobial properties. The novel combination was assessed for antimicrobial effects against a range of Gram positive and negative bacteria to develop novel stable formulations to combat drug resistant bacterial infections.

## Methods

### Preparation of pomegranate rind extracts

PRE were prepared by blending 15 grams of finely sectioned pomegranate rind with 45 mLs distilled water for 10 min. The crude extract was filtered through muslin followed by Whatman No. 1 filter paper prior to autoclaving (121°C for 15 mins) or filter sterilisation using a 0.2 μm filter (Millipore), before storage at -20°C [19]. A sample of the PRE was freeze-dried to determine the dry weight content which was found to be 0.0437 g/ml.

### Screening assay

Overnight cultures of the Gram positive strains *S. aureus *(NCTC 6571)*, B. subtilis *(NCTC 6452) and the Gram negative strains *E. coli *(NCTC 12241), *Ps. aeruginosa *(NCTC 950) and *P. mirabilis *(NCTC 7827) were prepared on nutrient agar plates (Oxoid Ltd, UK). All bacterial isolates (purchased from the National Collection of Type Cultures, Health Protection Agency, UK) were suspended in Ringer's solution (Oxoid, UK) to a turbidity equivalent to 0.5 McFarland (1.5 × 10^8 ^CFU/ml) and 100 μL were spread onto Mueller-Hinton agar plates (Oxoid Limited, UK). The PRE (10 μL) was then spotted onto sterile Whatman no 1 filter paper discs (5 mm diameter) placed centrally on the plates which were incubated at 37°C for 24 h prior to recording zones of inhibition. Penicillin G (1 unit, Mast Diagnostics UK) was used as a control.

### Antimicrobial activity of PRE/metal salt combinations

All reagents were purchased from Sigma-Aldrich (Poole, Dorset) and distilled water was used throughout. Overnight cultures on nutrient agar were prepared as previously described. An aliquot of PRE (330 μl) was added to 700 μl of the freshly prepared solutions (4.8 mM) of metal salts (FeSO_4_, CuSO_4_, MnSO_4_, ZnO) and the final solution was protected from light [19]. The appropriate bacterial dilution was prepared in Ringer's solution and 50 μl were placed in a sterile Eppendorf micro-centrifuge tube with a 100 μl aliquot of the PRE/metal salt solution. After exposure for 30 minutes at room temperature, the activity of the PRE/metal salt combination was neutralized by adding an equal volume of 2% (v/v) Tween-80 (Sigma Chemical Co., UK) in Lambda buffer [19]. Serial dilutions were prepared in Ringer's solution and 10 μl of each dilution was spotted onto nutrient agar plates and incubated for 24 hours at 37°C. Each assay was conducted in triplicate.

### Antimicrobial activity of PRE/metal salt combinations plus vitamin C

The assay was carried out as described above with the following addition: vitamin C was added to the metal salt (FeSO_4_, CuSO_4_) solution immediately prior to mixing with the PRE. Aliquots of vitamin C were added to give final metal ion: vitamin C ratios (and vitamin C concentrations) of 1:1 (4.8 mM), 1:5 (24 mM), and 1:20 (96 mM). A 700 μl aliquot of the solution of metal salt/vitamin C was then added to PRE prior to assay.

### Fractionation study

PRE was fractionated using Millipore ultra-filtration devices (nominal M. Wgt. cut-off = 5,000 a.m.u.) and the resulting extracts tested by the screening method described above.

### Statistical analyses

Statistical analyses of the results were carried out using SPSS (ver. 14) and the data were analysed by a one way Anova and Tukey's multiple comparison test.

## Results and discussion

Preliminary screening employing the disc diffusion assay was utilised to compare the antimicrobial activity of the autoclaved and filter sterilised extracts following incubation for 12 h and 24 h against a panel of bacteria. The PRE, sterilised by either method, exhibited antimicrobial activity at each time point against the Gram positive organisms (*S. aureus *and *B. subtilis*) (Table 1). The autoclaved extract demonstrated slightly greater activity than the filter sterilised extract; however, this increase was not statistically significant (P = 0.4). The Gram positive bacteria also demonstrated the largest zone of inhibition against 1 unit of penicillin G, with *S. aureus *revealing the largest zone of inhibition.

**Table 1:** Diameter of the zones of inhibition (mm) of the autoclaved pomegranate rind extract compared to penicillin and the results of the fractionation PRE against a panel of five bacteria.

**Organism**	**12 h**	**24 h**	**Penicillin G**	**Low Molecular Weight Fraction**	**Whole PRE fraction**
*S. aureus*	14	14	36	11 ± 0.25	14 ± 0.14
*B. subtilis*	10	10	32	13 ± 0.09	15 ± 0.14
*Ps. aeruginosa*	7	0	9	0	0
*P. mirabilis*	0	0	0	0	0
*E. coli*	0	0	0	0	0

For the Gram negative organisms, a small zone of inhibition (7 mm) was observed with *Ps. aeruginosa *at 12 h incubation with extracts from both sterilisation procedures; however, at 24 h no zone of inhibition could be detected with any Gram negative bacteria in the test panel (Table 1). This may indicate an inhibition of physiological processes that is overcome upon extended incubation. A zone of inhibition with penicillin G was only observed against *Ps. aeruginosa *and no inhibition was seen with either *P. mirabilis *or *E. coli*.

Meléndez and Capriles [7] tested the antimicrobial properties of a number of tropical plants from Puerto Rico using the disc diffusion method against *E. coli *and *S. aureus*. They demonstrated that pomegranate extract produced inhibition zone sizes of 11 and 20 mm, for *E. coli *and *S. aureus *respectively. Thus, their results contrast to the present study in that a smaller zone of inhibition for *S. aureus *was observed along with antimicrobial activity against *E. coli*. Nascimento *et al. *[20] prepared ethanol extractions of a number of plants and tested these against a range of laboratory and clinical isolates. Interestingly, this group only reported antimicrobial activity for pomegranate extracts against laboratory strains of *Ps*. *aeruginosa *and *B. subtilis*. In their test, *B. subtilis *produced a zone of clearing equal to or greater than 7 mm. These differences may in part be due to the different extraction methods employed, potentially the freshness of the fruit used, and variations in the season and region of growth.

Variations in results between studies on pomegranate extracts are not only seen in disc diffusion assays, but have also been recorded with minimum inhibition concentration (MIC) determinations. Values for MIC have been reported in a number of studies, ranging from 0.62–10 mg/ml against *S. aureus, E. coli and Ps. aeruginosa *[4] and up to 250 mg/l against *S. aureus *[21]. These differences could be due to the extraction method, freshness of the fruit, season and region of growth.

The antimicrobial activities of whole PRE/metal salt combinations were assessed using a modified version of the method adopted by Stewart *et al*. [19]. The PRE alone did not exhibit antimicrobial activity against any of the isolates, which may be due to the short incubation time of 30 minutes. However, the data from the previous disc diffusion assays demonstrate that if left for a longer period of time (12 and 24 h), PRE alone shows antimicrobial effects against three of the five bacteria tested, as has been shown previously. The metal salts alone showed a minimal amount of activity, with a reduction of *circa *10^1^–10^2 ^CFU/ml in cell population, however the greatest decrease (*circa *10^4 ^CFU/ml) (*p *< 0.5) was seen with *E. coli *upon addition of Fe (II), Cu (II), and Zn (II) ions.

**Figure 1:**
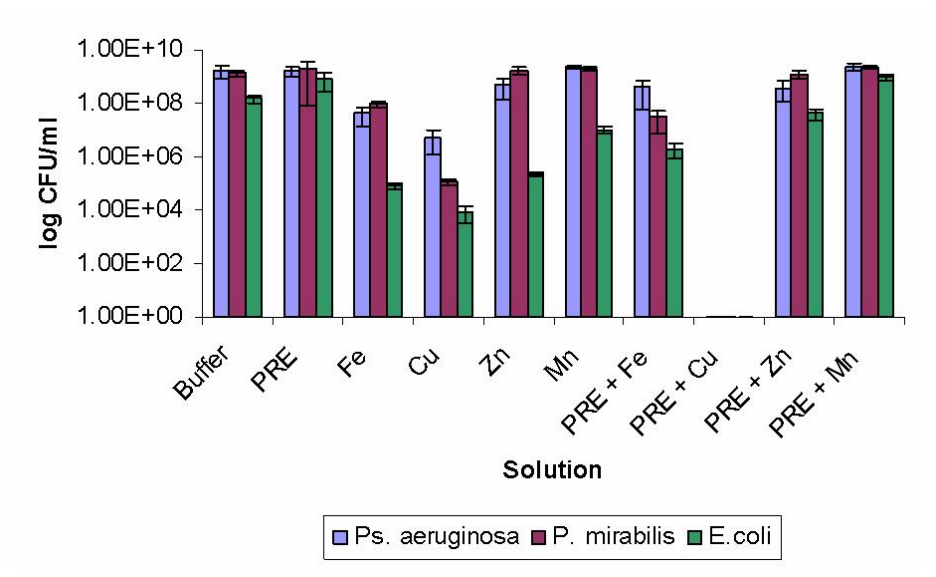
**The antimicrobial activities of PRE alone and in combination with metal ions against *Ps. aeruginosa, P. mirabilis *and *E. coli***. Lambda buffer was used as a control and error bars are SEM for each sample tested.

For the Gram negative bacteria the combination of PRE/Cu (II) resulted in no detectable growth for all three isolates after 30 mins (Fig 1), giving better results than the PRE or Cu (II) alone (*p *< 0.001). Conversely the combinations of PRE with the other metals salts were less active than the PRE and metal salts individually. A number of possible reasons exist for this observation; the PRE and other metal salts (Zn (II) and Mn (II)) may not have formed complexes. In addition, the lack of antimicrobial activity could be due to the PRE and Zn(II) or Mn(II) having formed a complex that owing to instability had little or no effect on the bacteria.

**Figure 2:**
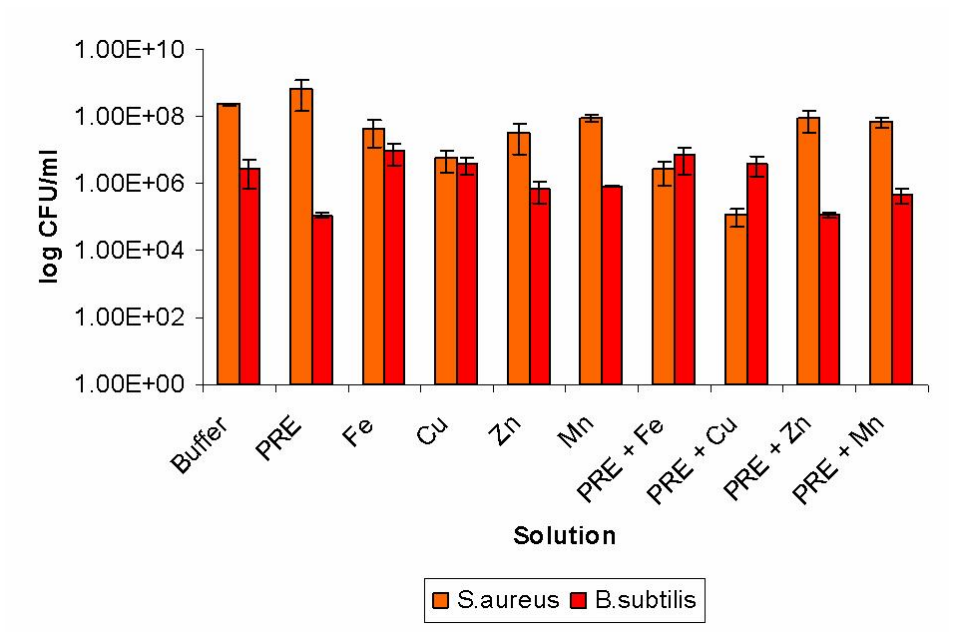
**The antimicrobial activities of PRE alone and in combination with metal ions against *S. aureus *and *B. subtilis***. Lambda buffer was used as a control and error bars are SEM for each sample tested.

Moderate antimicrobial activity was seen with the PRE/Cu (II) ions in combination against *S. aureus *reducing the surviving population by *circa *10^3 ^CFU/ml compared to the buffer (Fig 2). The remaining solutions of metal ions or their combinations with PRE only demonstrated minor or no antimicrobial effects against *S. aureus*. The difference in the results seen between the *S. aureus *compared to the Gram negative bacteria for the combination of PRE/Cu(II) is interesting. As the mode of action of this combination against bacteria is currently unknown; these results suggest that a thick cell wall, as present in the Gram positive bacteria, may inhibit the uptake/action of this potential antimicrobial combination.

Minimal differences between metal ions or combinations with PRE and controls were detected against *B. subtilis*, although a reduction of approximately 10^1 ^CFU/ml was obtained with PRE, the largest reduction seen in all the isolates (Fig 2). The limited antimicrobial effects seen with *B. subtilis *may be due to the high selectivity for up-take of metal ions in contrast to the non-specificity of its metal ion efflux systems [22]. However, some microorganisms have specific systems to remove metals from the cell whereas in *Bacillus *spp. copper is removed by a P-type ATPase protein [23].

A recent study investigated the antimicrobial affects of vanillin complexes with a number of different metal salts, against a range of bacteria [24]. Antimicrobial activity in this study was determined by agar diffusion and results showed the most active complex was vanillin and metals salts against the test bacteria (*S. aureus, E. coli, K. pneumaniae, P. vulgaris, P. aeruginosa, Candida albicans*). The current study and report by Sivasankaran and Selwin [24] demonstrate that antimicrobial properties of natural products can be enhanced by the addition of metal ions, especially cupric salts.

**Figure 3:**
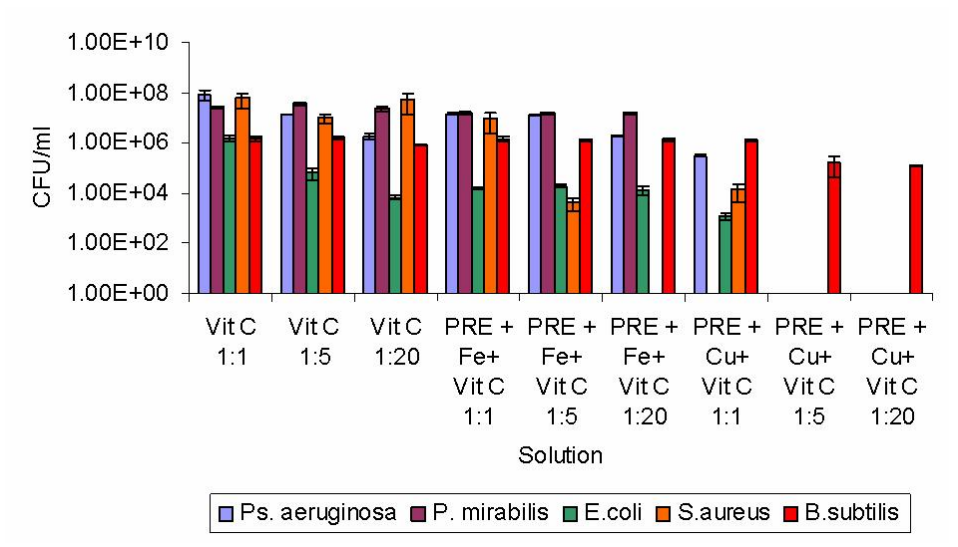
**The antimicrobial activities of PRE/metal ion combinations with the addition of vitamin C all isolates**. Lambda buffer was used as a control and error bars are SEM for each sample tested.

Further studies were conducted to prolong the activity of the PRE/metal combination by addition of the stabiliser vitamin C. Owing to the high activity exhibited by the PRE/Cu(II) combination against all Gram negative isolates, we studied the addition of vitamin C to the PRE/Fe(II) combinations (Fig 3). No enhancement of activity was recorded with two isolates, namely *Ps. aeruginosa or P. mirabilis*. For *E. coli*, a decrease in survival of 10^2 ^CFU/ml was seen with the addition of a stoichiometric equivalent of vitamin C (with respect to metal ion concentration). Further additions of vitamin C (to 5 and 20 equivalents) had no further effects on activity. Equally, no improvement in activity was observed for the addition of one equivalent of vitamin C to the PRE/Fe(II) combination against *S. aureus*; however, addition of 5 and 20 equivalents of vitamin C resulted in a reduction in growth of 10^3 ^CFU/ml and no discernable growth respectively.

For *S. aureus*, the addition of vitamin C to the PRE/Cu(II) mixture had no significant effect at one equivalent but a marked effect at 5 and 20 equivalents of vitamin C (no detectable growth in either). Addition of vitamin C to the PRE/Cu(II) solution had minimal effects on the survival of *B. subtilis*. Interestingly the addition of one equivalent of vitamin C decreased the efficacy of the PRE/Cu(II) with both *Ps. aeruginosa *and *E. coli*; however, further addition of vitamin C resulted in no recorded growth of both isolates. Further studies are required to investigate these effects.

In order to determine the approximate molecular weight fraction containing the active component(s) of crude PRE extract, it was subjected to fractionation using a molecular weight filter with a nominal 5,000 a.m.u. cut off. Similar activities were exhibited by the low molecular weight fraction and the intact whole PRE suggesting the antimicrobial component(s) of the PRE have low molecular weights. Analysis of the ultrafiltration device revealed that the high molecular weight fraction was negligible which in part is attributable to the PRE preparation methods.

## Conclusion

Combinations of PRE with Cu(II) ions exhibit enhanced antimicrobial effects against *E. coli*, *Ps. aeruginosa *and *P. mirabilis *and moderate activity is observed against *S. aureus *in comparison to each component. The addition of high quantities of vitamin C markedly enhanced the activities of both PRE/Fe(II) and PRE/Cu(II) mixtures against *S. aureus*. Future investigations into the activity of PRE/Cu(II) combinations against clinical isolates of *Ps. aeruginosa and S. aureus *are being conducted. The active component(s) in the PRE was found in the low molecular weight fraction and further work is underway to isolate it and to establish the mode of action, along with the mechanism of enhancement by metal salts and vitamin C.

## Competing interests

The authors declare that they have no competing interests. The study was in part funded by Nature Therapeutics Ltd.

## Authors' contributions

SWJG, MDF, AFK, WES, DPN participated in the design of the study. EMM, carried out the microbiological analysis. EMM, SWJG, MDF, AFK, DPN analysed the data and wrote the paper. All authors read and approved the final manuscript.

## Pre-publication history

The pre-publication history for this paper can be accessed here:


